# Supplementary figures and images for: Characterization of the Drosophila Adult Hematopoietic System Reveals a Rare Cell Population With Differentiation and Proliferation Potential
Source: Front Cell Dev Biol. 2021 Oct 13;9:739357. doi: 10.3389/fcell.2021.739357 (PMC8550105; doi:10.3389/fcell.2021.739357)

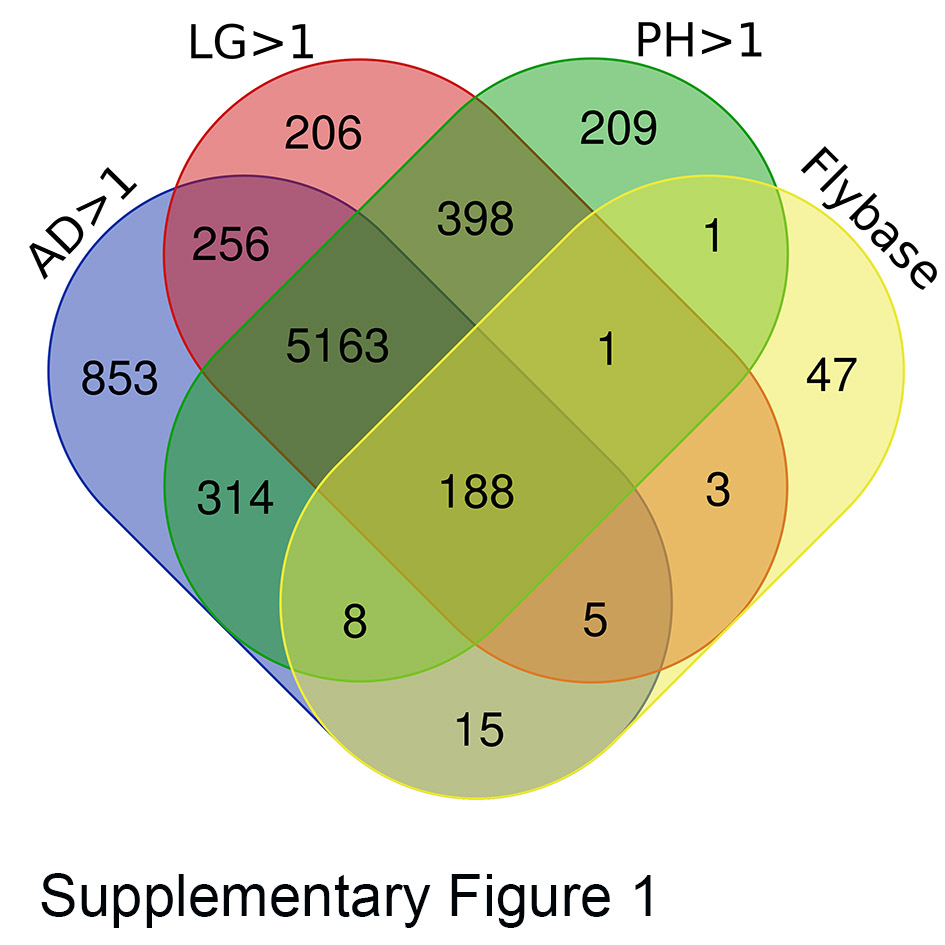

Supplement: Supplementary Figure 1 — Venn diagrams showing the number of genes expressed (RPKM > 1 in all three biological replicates) in adult hemocytes (AD), in larval peripheral hemocytes (PH) and in larval lymph glands (LG) as determined by RNA-seq on w1118 females, in comparison with those annotated as hemocyte markers according to Flybase. [file Image_1.JPEG]

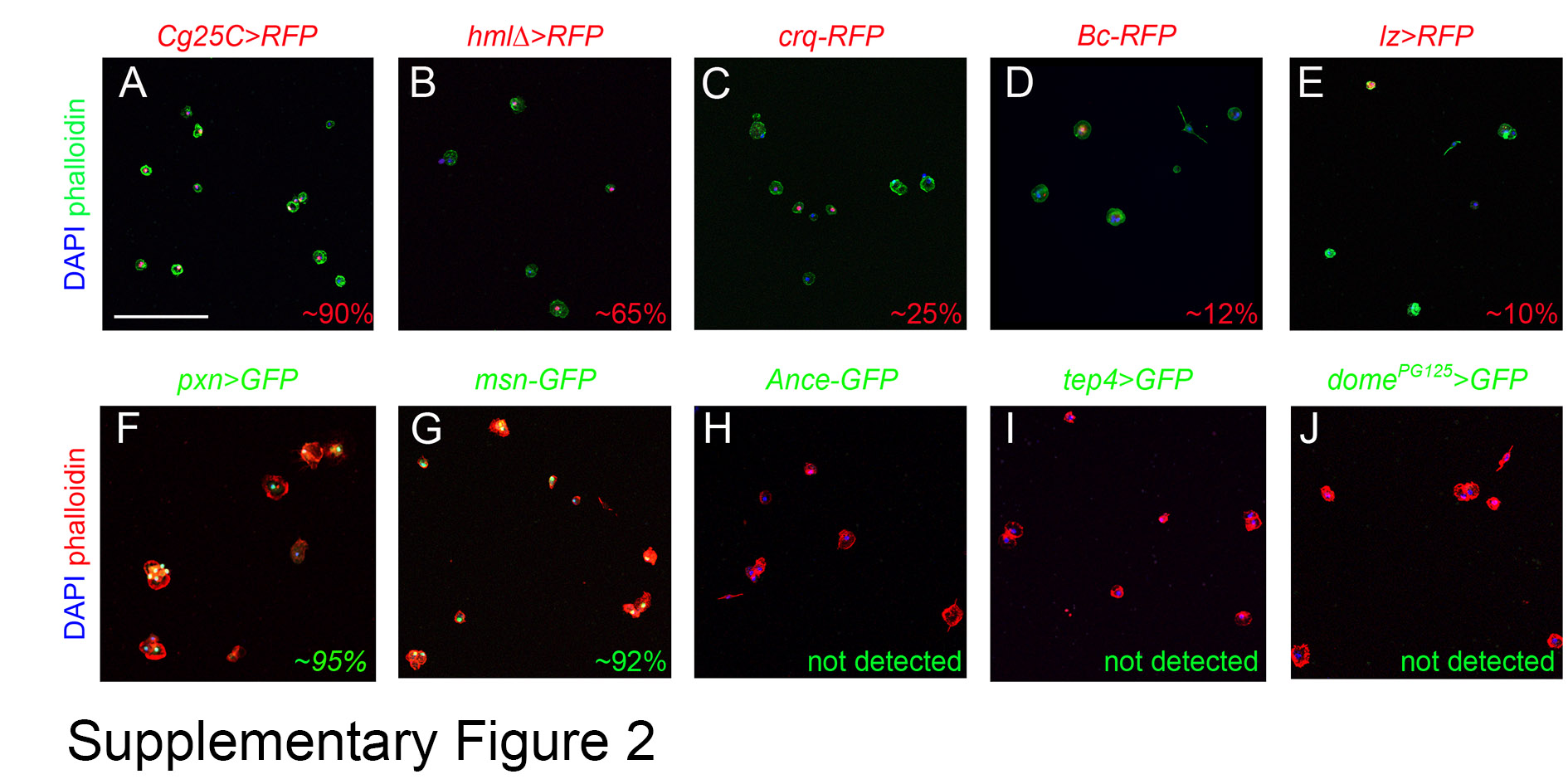

Supplement: Supplementary Figure 2 — Confocal views showing representative bleeds from 5-day-old flies carrying the following transgenes: Cg-GAL4, UAS-RedStinger (A, Cg25C>RFP), HmlΔ-GAL4, UAS-RedStinger (B, HmlΔ>RFP), crq-RFP (C), BcF6-mCherry (D, Bc-RFP), lz-GAL4, UAS-RedStinger (E, lz>RFP), pxn-GAL4, UAS-2xEYFP (F, pxn>GFP), msnF9-GFP (G, msn-GFP), Ance-GFP (H), tep4-GAL4, UAS-2xEYFP (I, tep4>GFP), domePG125-GAL4, UAS-2xEYFP (J, dome>GFP). Cells were counterstained with phalloidin (A–E: green, F–J: red) and DAPI (blue). Scale bar 200 μm. The percentage of cells expressing each transgene is indicated in the bottom right corner of each panel. [file Image_2.JPEG]

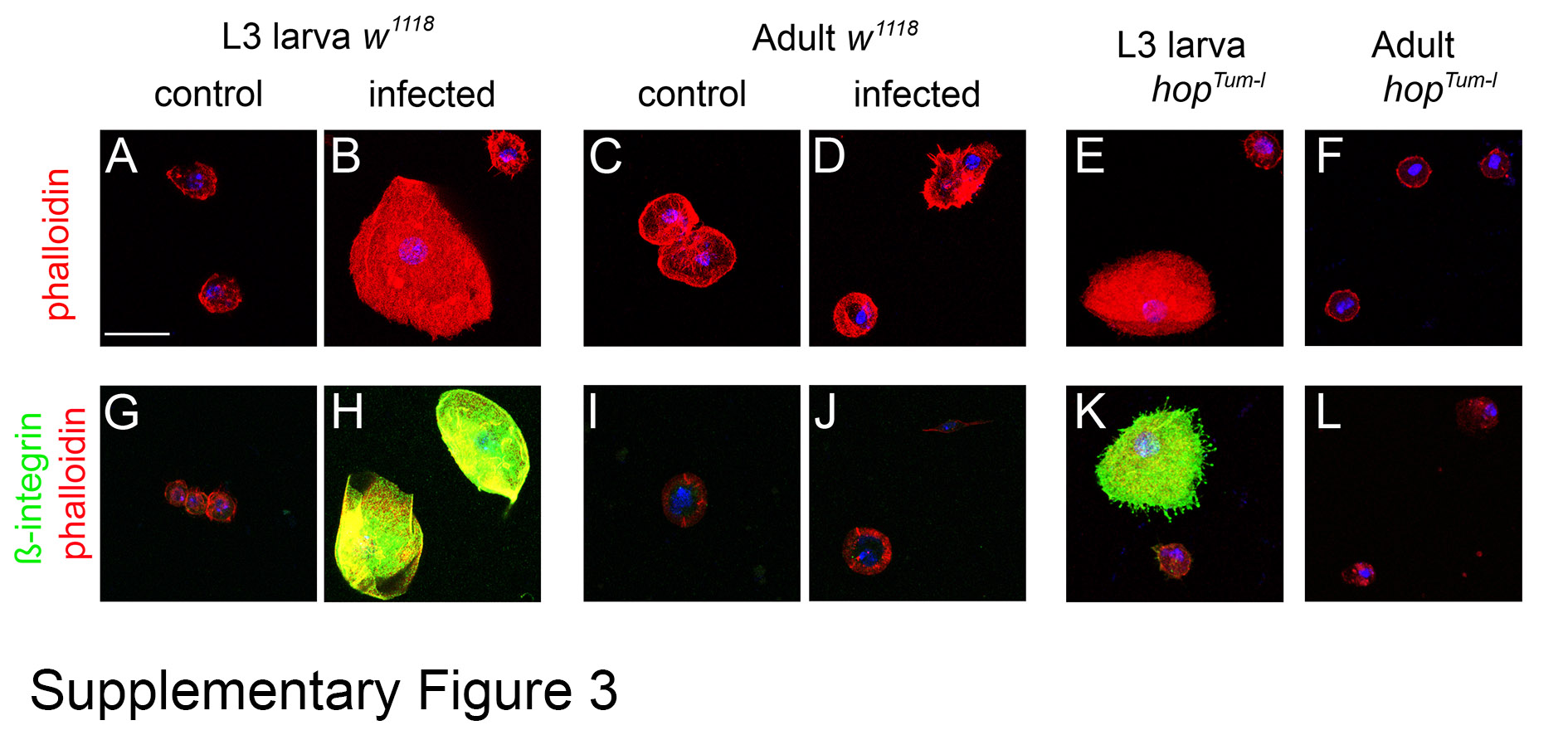

Supplement: Supplementary Figure 3 — Confocal views of larval (A,B,E,G,H,K) and adult (C,D,F,I,J,L) blood cells from control (A,C,G,I) or L. boulardi-infected (B,D,H,J) w1118 flies or from HopTum–l flies (E,F,K,L). (A–F) Blood cells morphology was revealed with phalloidin staining (red). (G–L) Blood cells were stained with phalloidin (red) and anti-β-integrin (green). (A–L) Nuclei were stained with DAPI (blue). Scale bar 20 μm. [file Image_3.JPEG]

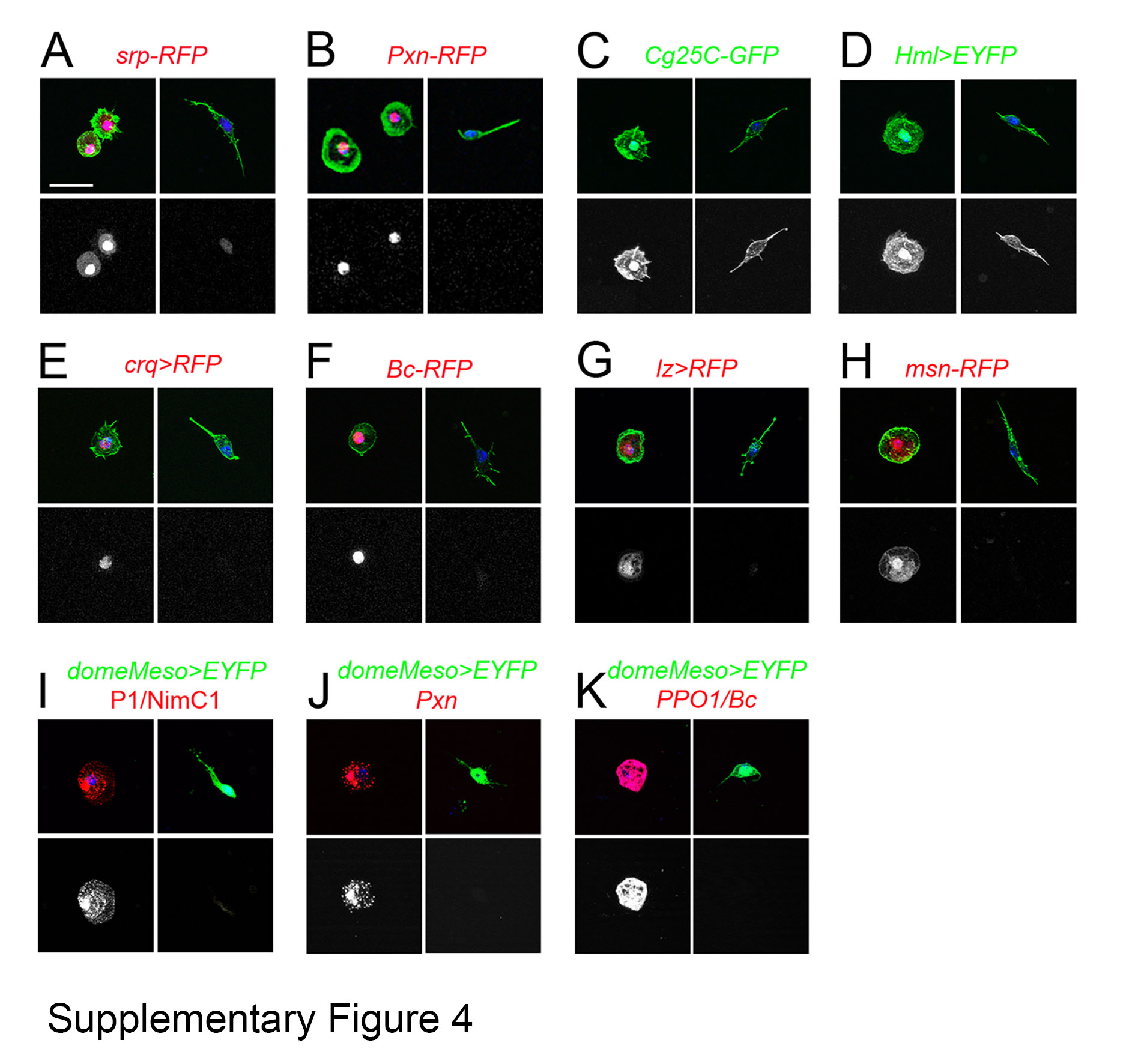

Supplement: Supplementary Figure 4 — (A–H) Confocal views of hemocytes from adult flies carrying the following transgenes: srpHemo-His2A-RFP (A), Pxn-RedStinger (B), Cg25C-GFP (C), HmlΔ-GAL4, UAS2xEYFP (D), crq-GAL4, UAS-RedStinger (E), BcF6-mCherry (F), lz-GAL4, UAS-RedStinger (G), msnF9-mCherry (H). Cells were counterstained with phalloidin (green) and DAPI (blue). Lower panels display the red (A,B,E–H) or green (C,D) channel only. (I–K) Confocal views of hemocytes from domeMeso-GAL4, UAS-2xEYFP adult flies following immunostaining against P1/NimC1 (I, red) or in situ hybridization against Pxn (J, red) or PPO1/Bc (K, red). Cell nuclei were stained with DAPI (blue). The lower panels display the red channel only. (A–K) Scale bar 20 μm. Left panels display round hemocytes and right panels show fusiform/domeMeso+ cells. [file Image_4.JPEG]

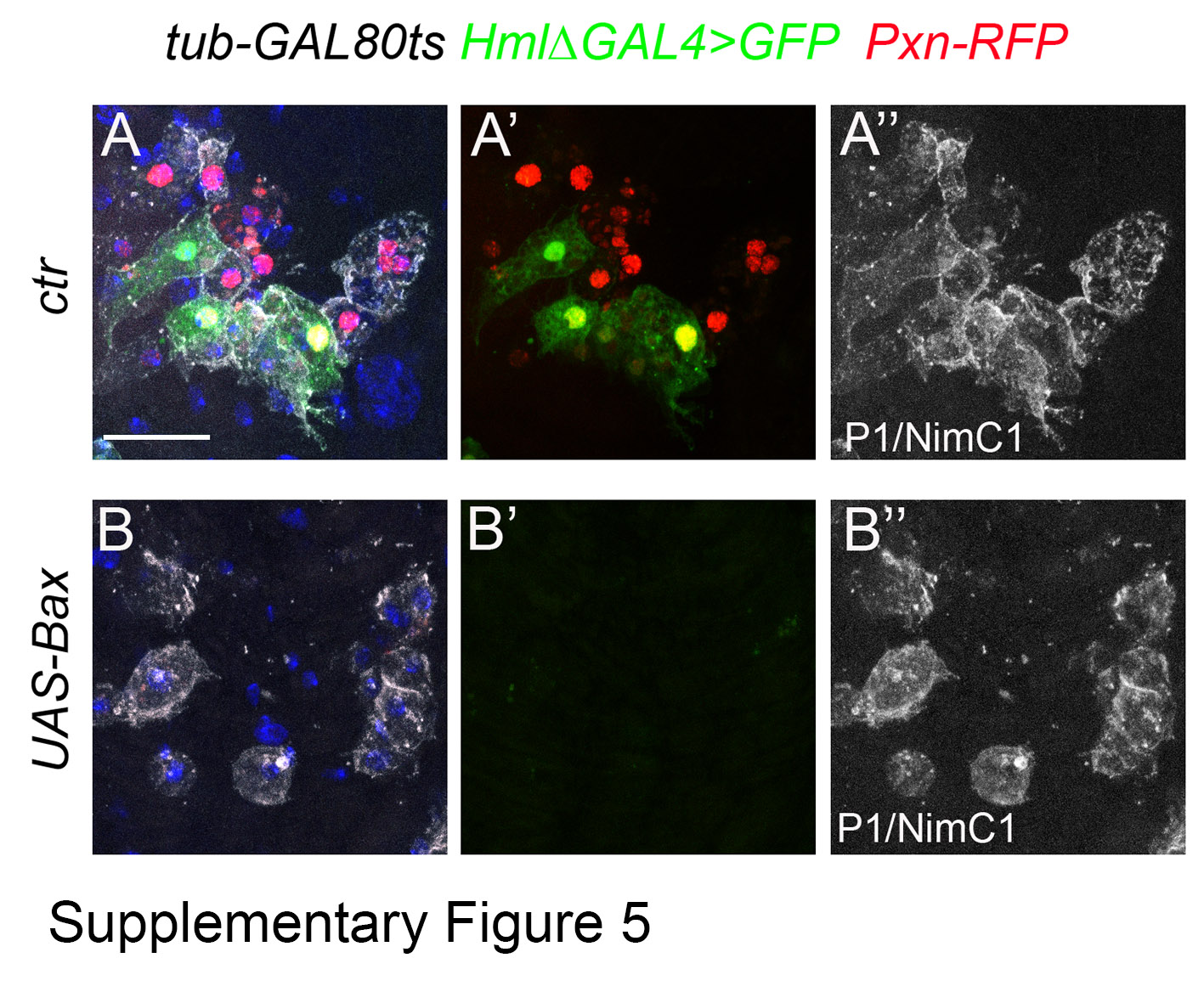

Supplement: Supplementary Figure 5 — Confocal views of abdominal hematopoietic hubs from tub-GAL80ts, Pxn-RFP, HmlΔ-GAL4, UAS-2xEYFP control (A) and tub-GAL80ts, Pxn-RFP, HmlΔ-GAL4, UAS-2xEYFP, UAS-Bax (B) flies raised at 29°C. Cells were stained with anti-P1/NimC1 (white) and DAPI (blue). (A’,B’): green (GFP) and red (RFP) channels. (A”,B”): white channel (P1/NimC1). Scale bar 20 μm. [file Image_5.JPEG]

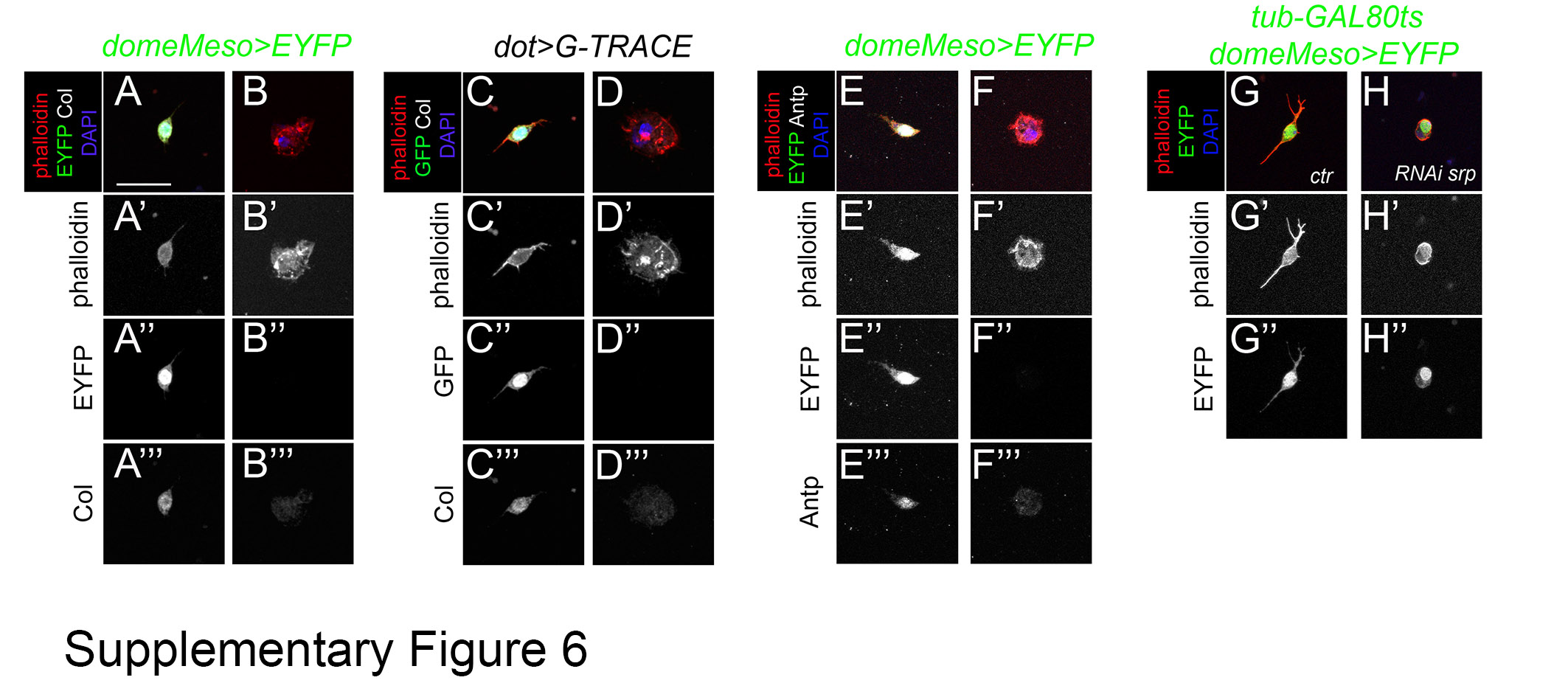

Supplement: Supplementary Figure 6 — (A–F) Confocal views of Col (A–D) or Antp (E,F) expression in fusiform (A,C,E) or round (B,D,F) hemocytes from domeMeso-GAL4, UAS-2xEYFP (A,B,E,F) or dot-GAL4, G-TRACE (C,D) adult flies. (C,D) G-traced (past) activity of dot-GAL4 is visualized by nuclear GFP expression. No present expression (nuclear RFP) was observed. (G,H) Confocal views of domeMeso-GAL4, UAS-2xEYFP cells in control (G, ctr) and UAS-RNAi srp (H, RNAi srp) adult flies. The knock-down of srp by RNAi prevents filopodia extension by domeMeso+ cells. Cells were stained with DAPI (blue) and phalloidin (red). Scale bar: 20 μm. [file Image_6.JPEG]

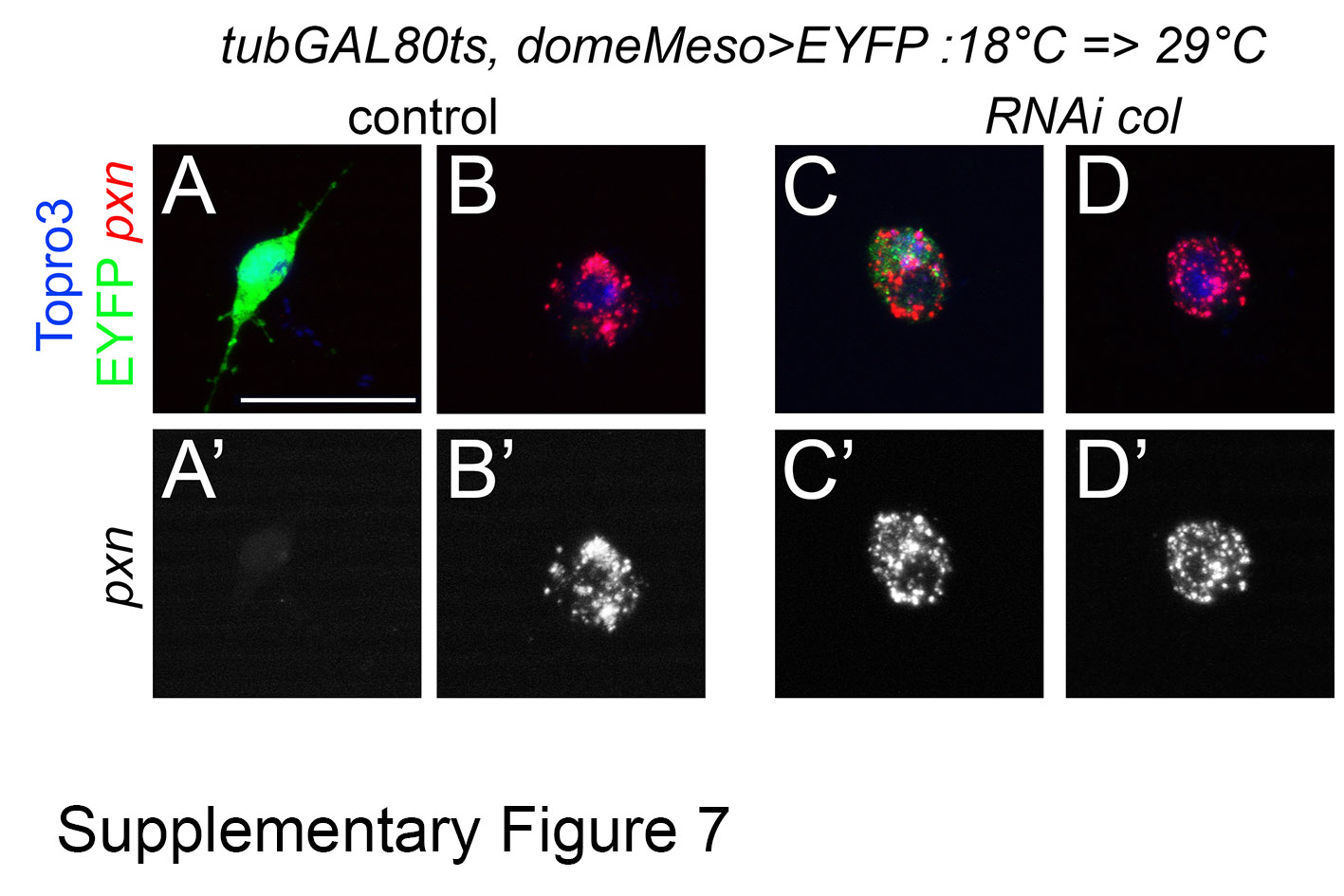

Supplement: Supplementary Figure 7 — Blood cells from tub-GAL80ts, domeMeso-GAL4, UAS2x-EYFP adult flies expressing (C,D) or not (A,B) col RNAi only during adulthood. Pxn expression (red) was visualized by in situ hybridization and EYFP (green) expression by immunostaining. Nuclei were stained with Topro3 (blue). The lower panels display the red channel only. Scale bar 20 μm. Left panels (A,C) show domeMeso+ cells and right panels (B,D) show domeMeso– cells. [file Image_7.JPEG]

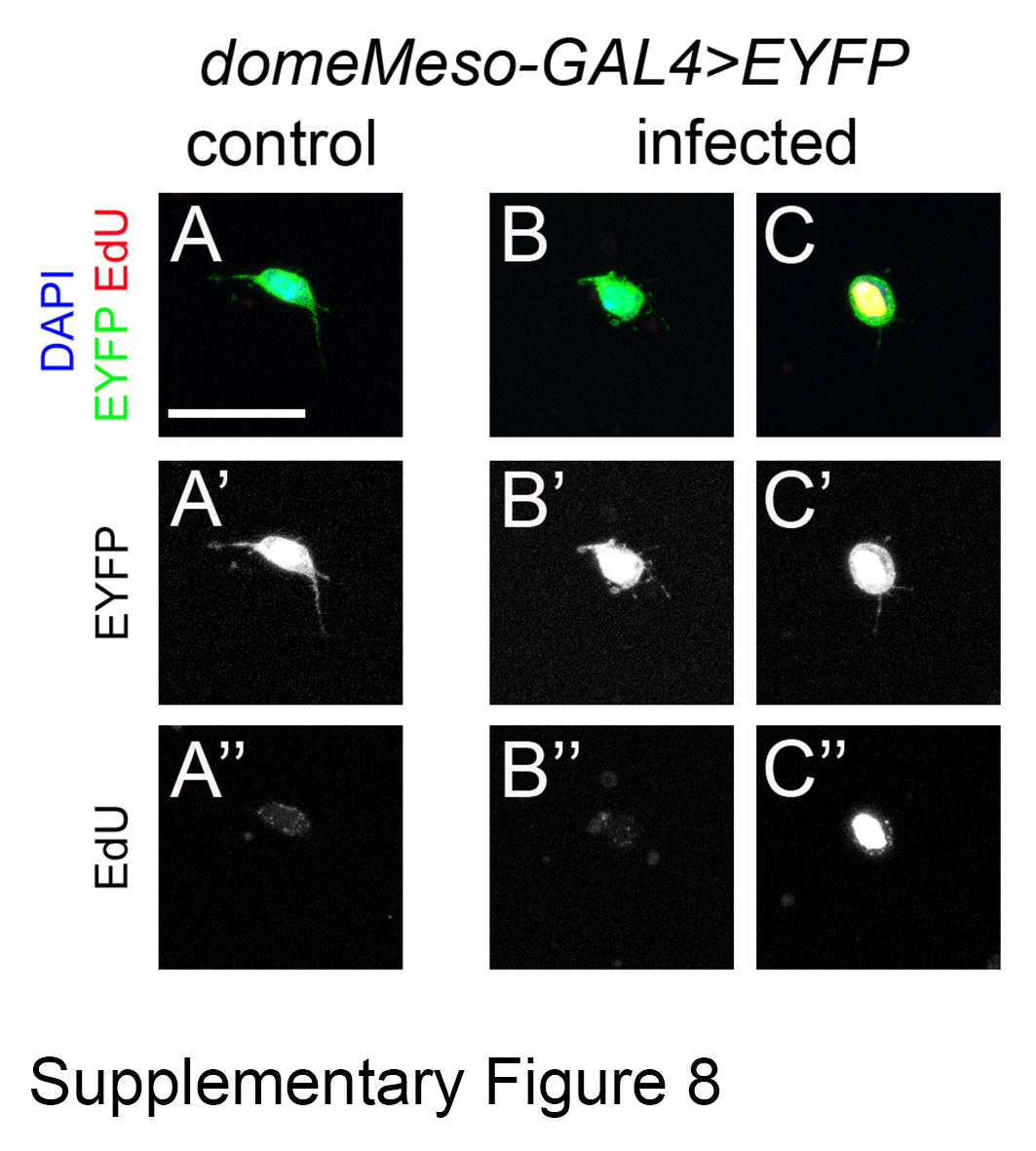

Supplement: Supplementary Figure 8 — (A–C) Confocal views of domeMeso-GAL4, UAS2x-EYFP blood cells from control (A) or E. coli-infected (B,C) adult flies fed with EdU. In vivo EdU incorporation was visualized in red. Nuclei were stained with DAPI. Scale bar 20 μm. The lower panels show only the green (A’–C’) or the red (A”–C”) channel. [file Image_8.JPEG]
